# A scoping review on the contribution of interprofessional collaborative practices on preventing and managing post-partum haemorrhage in the health care system

**DOI:** 10.1186/s12912-025-02988-z

**Published:** 2025-04-24

**Authors:** Nombulelo Esme Zenani, Patience Mashudu Tulelo, Khathutshelo Grace Netshisaulu, Nombulelo Veronica Sepeng, Maurine Musie, Rudwell Gundo, Fhumulani Mulaudzi

**Affiliations:** 1 NuMIQ Research Focus Area, School of Nursing Science, Faculty of Health Sciences, North-West University, Mmabatho, South Africa; 2 Department of Nursing, University of Pretoria, Pretoria, Republic of South Africa; 3 Department of Advanced Nursing Science, University of Venda, Thohoyandou, Republic of South Africa

**Keywords:** Adverse events, Interprofessional collaborative practices, Prevention, Maternal, Post partum haemorrhage

## Abstract

**Background:**

Postpartum haemorrhage (PPH) is a complication associated with increased morbidity and mortality. Effective management of PPH relies on the interdependent roles of various healthcare professions to ensure safe, high quality outcomes. This scoping review aims to explore and synthesise literature of the contribution of interprofessional collaboration in preventing and managing postpartum haemorrhage.

**Design:**

The review adopted a scoping review methodology underpinned by Arksey and O’Malley’s five stage framework. Four databases (CINHAL, Scopus, PubMed, and Medline) were searched for sources. The inclusion criteria consisted of full-text articles published in English between 2000 and 2024, across various research designs.

**Results:**

The review demonstrates that effective interprofessional collaboration has the potential to ensure prompt diagnosis and management of PPH, while also promoting positive patient and team dynamics outcomes. Moreover, interprofessional collaboration optimises resource utilisation and team synergy, with an awareness of each healthcare professional’s role, mutual trust and respect, and shared leadership.

**Conclusion:**

Preventing and managing PPH depends on highly effective interprofessional collaboration. To promote safe healthcare delivery during PPH emergencies, strengthening interprofessional collaboration practices is encouraged through various measures. These include interprofessional collaborative quality improvement initiatives, team-based PPH protocols, interprofessional situ simulation training, and PPH mock drills. These strategies provide healthcare systems with structures for better preparedness and swift interprofessional collaborative intervention to prevent complications of postpartum haemorrhage.

**Supplementary information:**

The online version contains supplementary material available at 10.1186/s12912-025-02988-z.

## Background

Every year, millions of women experience postpartum haemorrhage (PPH), defined as a blood loss of 500 ml or more within 24 h of birth [1]. Effective management of PPH requires prompt, timely, and interprofessional team collaboration to optimise the response to this maternal emergency and improve clinical outcomes. PPH prevention focuses on the third stage of labour, where the woman is provided with intravenous or intramuscular oxytocin, uterine massage, prompt blood transfusion for blood loss replacement, and umbilical cord traction [2]. Additional PPH management strategies include various medications to treat uterine atony, transfusion of blood products, tamponade techniques, uterine artery embolization, and surgical management [1]. Regardless of the indicated management strategies, a significant number of women globally suffer from PPH and require effective interprofessional collaboration (IPC) to manage PPH in the healthcare system and promote better patient outcomes in such maternal emergencies [3, 4].

According to the World Health Organization (WHO) [2], the incidence of PPH after vaginal delivery is 3.2% in the United States, 16% in Nepal, and 10.4% in China [1]. In low-income countries with limited resources, maternal deaths from PPH are higher, with more than 40% of maternal deaths in Africa attributed to PPH [5, 6]. In South Africa, 599 maternal deaths due to PPH were reported between 2020 and 2022, 86% of which could have been prevented. These deaths were attributed to delayed diagnosis of PPH by the interprofessional team, lack of skills in managing the maternal emergency and ineffective systems developed by the maternal interprofessional team to manage PPH. Prevention strategies include massive blood transfusion protocols for easy and prompt access to blood products, and the availability of PPH boxes in the facilities, including intravenous fluids, oxytocin, tranexamic acid, blood collecting tools, internal tamponade, and blood products [7]. Ramavhoya et al. [8] state that in Limpopo province, South Africa, PPH is the second leading cause of maternal deaths, accounting for 38.2% of maternal deaths reported at primary healthcare facilities. Contributing factors to this phenomenon include inadequate staffing of midwives, substandard care provided by midwives due to time pressures, and failure to identify PPH-risk patients. Moreover, pregnant woman in the low resource facilities often avoid disclosing previous histories of PPH due to fear of being transferred to high-risk clinics, which can involve traveling costs they cannot afford. A shortage of ambulances when in need of transferring patients to tertiary hospitals further increases the risk of fatalities among woman experiencing PPH in low resource facilities. The same authors further allude that there is poor interprofessional communication between the staff regarding patients as well as failure to disseminate information in time, hindering the implementation of guidelines for managing PPH [8].

To mitigate the abovementioned challenges, the [9] recommended that both low-resource and developed countries identify strategies to achieve the Global Sustainable Development Goals (SGD) maternal mortality target of less than 70 deaths per 100,000 live births by 2030. A number of strategies can be implemented such as including patients in the management of PPH through sensitising mothers to visit antenatal services, reporting a previous history of PPH, managing chronic illness such as hypertension during pregnancy, involvement of non-profit organisations that cater to the needs of childbearing woman in rural settings and most importantly improving IPC in obstetric care. The provided strategies aim to accelerate the reduction of PPH complications. Affirm that interprofessional care in managing PPH can decrease mortality rates related to complications of PPH in low-resource countries through the use telemedicine with remote consultation for high-risk patients, sharing of expertise, guidelines or possibly remote direction of certain procedures. Task shifting and sharing is acknowledged as the best solution for managing PPH in rural areas, provided there is proper training, clear guidelines, checklists, and referral indications. Such strategies can standardise care and patients who are at risk of PPH can have access to health care promptly.

Included in the interprofessional collaborative care practices (IPCP) strategies is PPH interprofessional team mock drills. Interprofessional PPH team mock drills must be routinely conducted for emergency preparedness, to strengthen team cohesion, and to raise awareness of roles and responsibilities when managing PPH as a team [7]. Contributing factors to poor care and PPH complications include a limited number of trained midwives and gynaecologists who portray high expertise and experience in managing PPH, as well as a lack of skills, such as using the partogram to identify prolonged labour and implementing active management of the third stage of labour, where the mother must be provided 10 units of Oxytocin [7]. It is crucial to note that prolonged labour is a risk factor for PPH due to uterine atony, a condition where the uterus fails to contract fully after delivery of the baby. This condition puts the woman at a higher risk of developing PPH. Therefore, midwives and the rest of the interprofessional obstetric team need to be skilled in partogram use and be competent in the interpretation of the partogram for early identification of prolonged labour and prompt intervention.

Delays in the active management of the third stage of labour increases the risk of PPH. Active management, which involves administering oxytocin immediately after birth to promote contraction of the uterus, is the most efficient approach to prevent PPH. Therefore, prompt active management of the third stage of labour is important to minimise PPH incidence. In case of an emergency caesarean section, preventing complications such as unplanned hysterectomy or hypovolemic shock (which can be fatal and lead to cardiac arrest from PPH), requires close consultation between the attending midwife, the gynaecologist, the surgeon on call, or the anaesthetists in the facility. Therefore, effective interprofessional communication and collaboration are of great importance in managing, as well as preventing PPH. PPH is a challenge in rural settings with limited access to the expertise and resources. This gap highlights the need for comprehensive interprofessional training across all levels of care, including training local birth assistants who do not have medical qualifications. Thus, an awareness of an evidence-based practice set of guidelines for managing PPH as per the WHO guidelines to all who assist women in childbirth [2].

**Fig. 1:**
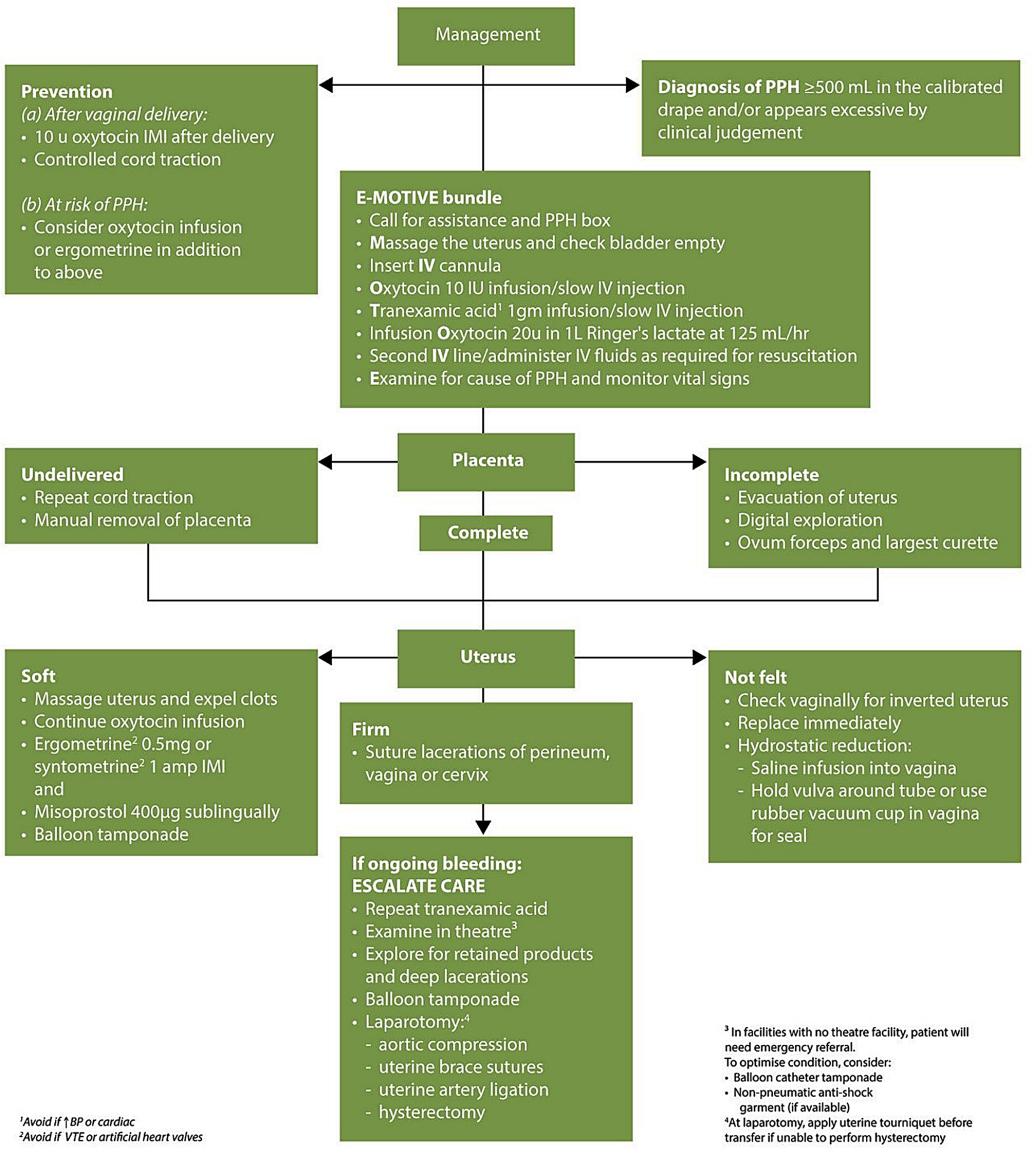
Postpartum haemorrhage (PPH) after vaginal delivery: Essential Steps in Managing Obstetric Emergencies (ESMOE) plus E-MOTIVE. (*IMI* intramuscular injection; *IV* intravenous; *BP* blood pressure; *VTE* venous thromboembolism.) accessed at: https://journals.co.za/doi/full/10.7196/SAMJ.2023.v113i12.1164

In the context of PPH, where there is a high risk of rapid deterioration in maternal and foetal status due to uncontrolled haemorrhage, healthcare facilities must initiate well-coordinated, team-based interventions. The interprofessional team in maternal care needs to possess advanced life support knowledge, skills, and values, along with clearly defined roles and responsibilities of interprofessional team members during PPH emergency response [7]. These roles include a predetermined team leader to facilitate the entire response, a person responsible for haemodynamic line placement, a team member to commence and lead volume resuscitation, another person to administer medication, one to perform uterine massage, a team member for documentation, and an additional runner [10]. In a significant breakthrough in managing PPH in low-resource countries, Singata-Madiki et al. [6] shared an interprofessional initiative that embedded the WHO recommendations for managing PPH. Figure 1 below narrates the intervention. The interprofessional intervention reduced PPH after vaginal deliveries by 60% in Nigerian, Kenyan, Tanzanian, and South African hospitals. However, it remains a trial and is not included in the national guidelines for PPH. The intervention can be initiated by trained midwives in collaboration with medical professionals. However, the authors highlighted that effective IPC, with an interprofessional team facilitating the intervention, would allow the bundle to be administered simultaneously and with better results [6]. Therefore, this beacon of hope, which calls for IPC, brings to a quest of exploring how best we can learn, work with, from, and about each other to optimise PPH management, especially within low-resourced areas.

Therefore, the above discussion highlights the need to strengthen interprofessional collaborative practices among healthcare professionals who attend to PPH maternal emergencies. The WHO defines interprofessional collaborative practice as a collaborative practice which occurs when multiple health workers from different professional backgrounds work together with patients, families, carers, and communities to deliver the highest quality of care across settings [11]. To mitigate the implications of PPH, interprofessional collaborative practices must be progressively explored and promoted. Moreover, the WHO has suggested IPC practices to promote a culture of patient safety, which includes creating systems for robust prevention, prompt identification, diagnosis, and management of PPH in the healthcare system. These measures align with the attainment of Sustainable Developmental Goal 3 which aims to ensure good health and well-being [2]. PPH can be prevented and managed using various IPC practices. One such IPC practice for managing PPH [6], interprofessional in-service simulation training, has been identified as a crucial for nurses, midwives, and medical doctors who work in maternity settings [3, 5, 12–15]. Research emphasises that receiving interprofessional simulation training could foster effective teamwork by ensuring that all team members have a comparable level of clinical understanding of PPH care bundles, along with the skills and values to comprehensively prevent and manage PPH [3, 5, 6, 12–15].

Interprofessional simulation training is increasingly recognised as an effective method for improving learning and performance to improve patient safety. To bridge the gap between simulation and reality, simulation teams should be representative of real-life teams, including various medical specialities and levels of expertise. Therefore, the use of real-life case scenarios, combined with the inclusion of an interprofessional team with interactive high-end mannequins, will be of great value for theory-practice integration and skills retension [16]. Intentional efforts to collaborate across disciplines are another form of IPC for PPH prevention and management. Collaboration across disciplines, such as midwives, obstetrics, anaesthetists, and other healthcare professionals, to develop a shared vision of care for women with PPH and streamline processes may necessitate project leaders understanding the different perspectives and goals of various departments and professionals [12].

The discussed strategies further strengthen interprofessional competencies, which are key to promoting positive patient outcomes in healthcare, particularly in managing maternal emergencies such as PPH [2]. Interprofessional competencies encompass four key domains. The first competency domain, shared interprofessional values and ethics, emphasises the need for a climate of shared values, ethical conduct, and mutual respect among the team managing women during childbirth. The second competency domain is awareness of the roles and responsibilities of the team. This implies that the team is able to use each other’s expertise to meet patient needs. The third competency domain, effective interprofessional communication, promotes responsible, respectful, and clear information shared compassionately within the team. The fourth competency domain, teamwork, emphasises that a team is expected to work together and have an awareness of team dynamics [11]. The discussed interprofessional competencies are the foundation of effective interprofessional collaborative practices. If effective interprofessional collaborative practices facilitate safe patient care and reduce mortality and morbidity rates, studies should be conducted to inform policy and practice for the best evidence-based practices. Thus, this review aims to map and summarise evidence on the role of interprofessional collaborative practices in preventing and managing PPH in the healthcare system. The review seeks to answer the following research question ***“What is available in the literature on the contribution of interprofessional collaborative practices in preventing and managing partum haemorrhage in the healthcare system”***

## Design

A scoping review was conducted according to the five-step framework proposed by Arksey and O’Malley [17]. The five steps followed include:

1. Identification of research objective
2. Search/identification of applicable studies
3. Selection of relevant studies
4. Charting of the data and
5. Collating, summarising, and reporting of the results

The scoping review methodology was found suitable for this study to map the literature on the topic and further identify gaps that can be addressed in future studies. The scoping review encompassed a broad range of literature examining the contribution of interprofessional collaborative practices in preventing and managing post-partum haemorrhage in the healthcare system. The PRISMA extension for Scoping Reviews (PRISMA-ScR) checklist was used to report the findings.

### Step 1: Identification of research objective

The research objective for this study was to explore and synthesise literature regarding the contribution of interprofessional collaboration in managing postpartum haemorrhage in the healthcare system. The scoping review question was guided by the PICO framework, which includes Population, Intervention, Comparison (not applicable in this study), and Outcome. The framework also informed the eligibility criteria for the scoping review.

#### Population

Interprofessional birth teams/maternal or child healthcare professionals. This team can consist of midwives, physicians, gynaecologists, or community/traditional birth assistants.

#### Intervention

Interprofessional collaboration practices in maternal care.

#### Comparison

Not applicable.

#### Outcome

Effectively prevented and managed postpartum haemorrhage occurrences.

### Step 2: Search applicable studies

The authors searched for articles in the following databases: CINHAL, PubMed, Medline, and Scopus. The search terms were level keywords: 1: interprofessional collaboration OR healthcare teamwork OR interdisciplinary teamwork OR obstetric teamwork OR interprofessional education OR interprofessional relationships OR prenatal care collaboration OR postnatal care teamwork OR interprofessional training OR health care coordination OR interdisciplinary communication OR collaborative health care models OR obstetric care teamwork. 2: AND postpartum haemorrhage prevention OR maternal health OR obstetric care OR maternal mortality OR perinatal care OR patient safety in obstetrics OR obstetric emergencies OR maternal outcomes. 3: AND haemorrhage OR rapid blood loss OR bleeding OR bleed OR uncontrollable bleeding. Ending on the 16 January 2024, the search was conducted by two of the authors in collaboration with a research assistant at the Health Science Faculty of the North-West University.

### Step 3: Selection of relevant studies

In this step, the authors decide on the inclusion and exclusion criteria for the studies. The criteria were based on the specifics of the research question and aligned with the subject matter by becoming familiar with the obtained studies [18]. The inclusion and exclusion criteria of the review are listed below.

#### Inclusion criteria

This study included peer-reviewed qualitative, quantitative, mixed-method, and multi-mixed-method studies, as well as reviews and unpublished dissertations. Empirical studies were included to validate multiple hypotheses and increase knowledge on the topic. While reviews provided the authors with summaries of the state of research, the gaps, and recommendations for future studies concerning the research question of the scoping review. Grey search data were retrieved using PubMed and CINHAL, considering the PICO framework adopted for the review and checking the reference list of the identified articles. The timeframe for the included studies was between 2000 and 2024, with the rationale of reflecting the period of the commencement of the Millennium Development Goals to present, allowing for an assessment of the progress in minimising PPH through interprofessional practices. The study only included peer-reviewed articles that discussed interprofessional interventions, such as interprofessional training interventions, interprofessional quality improvement initiatives, and interprofessional guidelines which included interprofessional teams managing or preventing PPH in healthcare systems. These intervention settings could be hospital, primary healthcare, or public or private sector settings.

#### Exclusion criteria

We excluded non-peer-reviewed articles, reports, and peer-reviewed articles published in languages other than English to ensure understanding and comprehension among the research team. Furthermore, all sources that did not meet the eligibility criteria of the PICO framework of the scoping review were excluded. Thus, all studies outside the maternal care setting, where the scope participants were not interprofessional, the intervention was mainly for one profession, or did not include interprofessional intervention or measures to prevent and manage PPH, were excluded. These exclusions ensured that only studies which answered the research question, *“What is available in the literature on the contribution of interprofessional collaborative practices in preventing and managing partum haemorrhage in the healthcare system”* were included.

**Fig. 2:**
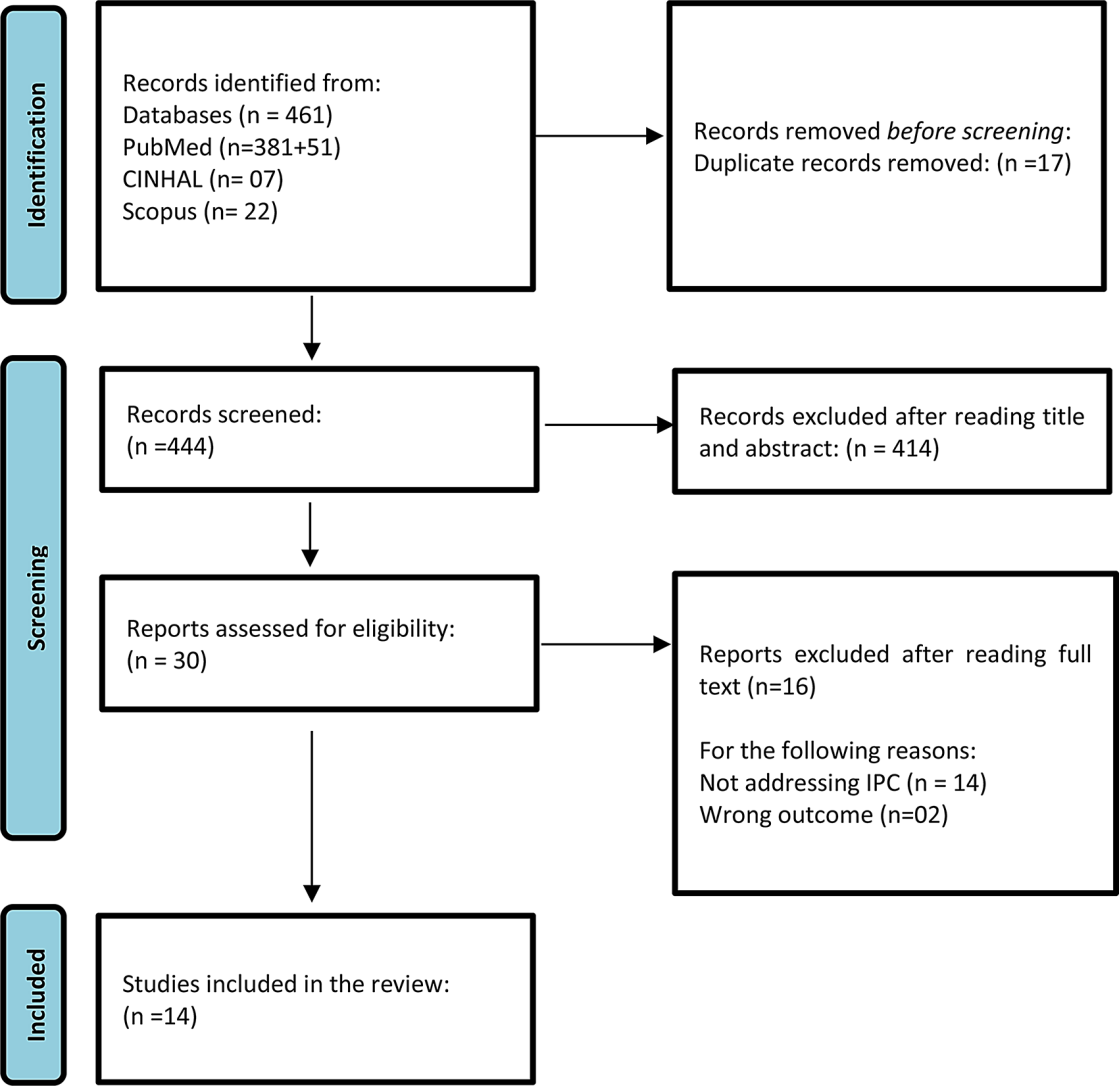
PRISMA flow diagram describing the screening and selection process, adapted from the 2020 PRISMA flow diagram

To manage the citations, all sources were imported into a software-based reference management system, Rayaan. The articles were screened by two of the authors, with any disagreements resolved through consensus among all the authors. The articles were screened first by title, then by abstract, and finally by full text. Duplicates were deleted, and sources which did not meet the eligibility criteria were excluded. The figure below represents the data search strategy using a PRISMA diagram (Fig. 2).

### Step 4: Charting of the data

In this step, the authors developed a data chart form to extract data from the included studies. The table below provides a descriptive analysis of the included studies. Two authors charted the data separately, compared the findings, and collaboratively revised and finalised the chart (Table 1).

**Table 1:** Data extraction with the included sources

Author/s, year of publication & country	Research methodology	Population	Interprofessional intervention	Impact on managing pph
Brazil et al. (2022) [3], Australia	Mixed Method research (collaborative action research approach)	Junior and senior midwives, junior and senior obstetricians, and anaesthetic doctors	Three types of collaborative interventions were implemented. These were relational interventions aimed to enhance team relationships; process interventions focused on redesigning the work process to achieve positive outcomes; and structural interventions targeted teamwork training, shared accountability and shared information systems	The IPC model gave productive teamwork and conflict management techniques a deeper meaning. Major PPH cases decreased significantly in the labour ward
Greer et al. (2023) [15], USA	Prospective, multisite, cohort study	Obstetrics and gynaecology attending physicians and residents, anaesthesia attending physicians and residents, certified and student nurse anaesthetists, paediatric attending physicians and residents, family practice attending physicians and residents, certified nurse midwives, obstetric and neonatal nurses, Navy hospital corpsmen (who function as medical assistants), blood bank personnel, and operating room personnel	Team training on PPH treatment and maternal and neonatal resuscitation	The multidisciplinary team improved in teamwork and PPH protocol adherence after the OB-STaT program
Wiesehan et a. (2023) [19], USA	Evaluative study (Economic analysis)	Patients’ costs records and Nurses in managerial positions who implemented or maintained the PPH–SMM reduction initiative	Training on the implementation of the PPH toolkit to prevent SMM	Implementing the PPH–SMM reduction initiative reduced the possibility of complications from PPH and reduced emergency hysterectomy
Brogaard et al. (2019) [12], Denmark	Observational study (Video review)	Obstetric teams (Obstetricians and Midwives) managing real-life postpartum haemorrhage	This was a purely observational study without any intervention, but the authors recommended IPC training on non-technical skills	No results (No IPC model was implemented after the study), but the study recommends continuous IPC and evaluation of teams to prevent PPH
Lutgendorf et al. (2024) [20], USA	Multi-site before-and-after study	Electronic medical records of patients who delivered between February 2018 and November 2019	Interprofessional obstetric teamwork training	Training did not have a significant effect on the decrease of PPH, but there were lower rates of massive transfusions and the transfer time for PPH was shortened. Management of PPH improved after training
Nelissen et al. (2017) [21]	Prospective educational intervention study	Clinicians, nurse-midwives, medical attendants (nurse aides without formal medical education) and ambulance drivers (without formal education)	Obstetric simulation-based training in the management of PPH	Following the training programme, there was a significant reduction in the incidence of PPH
Lutgendorf et al. (2017) [22], USA	Quality improvement project	Obstetric providers: obstetric staff/residents’ midwives, labour and delivery nurses, corpsmen and the remainder were anaesthesia providers	High fidelity, multidisciplinary in situ simulation training on the management of PPH	The collaborative training sessions assisted participants in improving teamwork when managing PPH. Communication and documentation also improved
Dillon et al. (2021) [16], USA	Prospective observational study	Nursing, obstetrical, and anaesthesia providers	PPH simulation training program	Post-partum haemorrhage improved following the implementation of a multidisciplinary simulation program
Egenberg et al. (2017) [13], Tanzania	Quasi-experimental, pre-vs. post-interventional study	Nurses, midwives, doctors, and medical attendants	Maternity staff simulated training on PPH	Improved teamwork during emergencies and communication resulted in a higher quality of maternal care. There was a significant reduction in the whole blood transfusion rate after training
Egenberg et al. (2017) [23], Tanzania	Descriptive and exploratory design	Nurse midwives, doctors, and medical attendants	Multi-professional simulation training on PPH	The multi-professional training resulted in improved management of clinical PPH
Bittle et al. (2018) [24], USA	Online didactic modules in combination with an interdisciplinary skills program	Registered nurses, obstetric and family medicine attending physicians and residents, advanced practice nurses, and ancillary staff	Multidisciplinary online theory and skill training program on the management of PPH	The program was effective in improving interdisciplinary team responses to PPH
Bell (2017) [25], USA	Retrospective chart review	Obstetric nursing leaders; obstetric physician leaders; staff from the blood bank, pharmacy, and simulation centre; and women who gave birth at these facilities	Multidisciplinary training on the implementation of PPH protocols and simulation training on the recognition and management of PPH	Multidisciplinary teams with nursing and physician champions were established, and obstetric haemorrhage and massive transfusion protocols were formed. Decrease in the number of women who had unplanned peripartum hysterectomies because of PPH

### Step 5: Collating, summarising, and reporting of the results

In this step, the authors collated and summarised the results and drew conclusions from the review [18]. The authors analysed the results using thematic analysis, and after reaching a consensus on the themes and subthemes, a descriptive discussion was conducted.

## Results

Three themes, each with subthemes, emerged from the review after the thematic data analysis. Namely, (1) Promotes prompt diagnosis and management of PPH; (2) Promotes patient safety and quality standards in managing PPH, and lastly (3) Enhances a positive and effective interprofessional team collaboration in managing PPH. The table below presents a narrative description of the themes and subthemes (Table 2).

**Table 2:** Description of the themes and the subthe

Theme	Sub-themes
Promotes prompt diagnosis and management of PPH	• Early identification and diagnosis of patients at risk (antenatal & intrapartum stage) • Fast administration of uterotonic medication and blood transfusion
Promotes patient safety and quality standards in managing PPH	• Promotes adherence to clinical protocols for the management of PPH • Positive patient outcomes
Enhances positive and effective interprofessional team collaboration in managing PPH	• Clear roles and responsibilities in managing PPH as a team • Promotes relational coordination in the team that manages PPH • Promotes accurate timelines of interprofessional communication among obstetrics team

### Theme 1: Promotes prompt diagnosis and management of PPH

The adoption of interprofessional collaborative practices in the healthcare system plays a significant role in promoting the prompt diagnosis and management of PPH [14]. According to Hernández et al. [14], IPC facilitates efficient coordination and communication among medical professionals, including nurses, midwives, doctors, and other team members. Studies on interprofessional team simulation and training demonstrate that collaborative practices can enhance prompt diagnosis and management of PPH [12–14]. This review highlights two significant subthemes related to the promotion of prompt diagnosis and management of PPH. The first subtheme related to the early identification and diagnosis of patients at risk of PPH, and the second was associated with the fast administration of uterotonic medication and blood transfusion.

#### Early identification of patients at risk and timely diagnosis of PPH

While PPH is apparent during labour, some risk factors, such as multiple pregnancies and a previous history of PPH, are identifiable during the antenatal care (ANC) period. A recent study by Munro et al. [26] asserted the importance of intensive training of healthcare professionals in improving the identification of patients at increased risk of PPH. This finding is corroborated by a prospective observational study by Nicolaides [27], which reported a marked improvement in blood volume estimation following onsite interprofessional training of obstetric personnel. A study by Brazil et al. [3] in Australia, which included an obstetrics interprofessional teamwork course, showed that simulation training, short practice sessions, and short videos increased the insight of the maternal team in the early identification of patients that pose a risk of PPH and received prompt iron supplements, were referred for structured care, and were timely diagnosed through the interprofessional handover process between the medical and midwifery teams. During the interprofessional handover process, the team reviewed the patient’s dataset, such as the vital data and partograms, shared knowledge, and collaborated to diagnose the patient. This study was further confirmed by Lutgendorf et al. [20] in the United States, who included interprofessional obstetric simulation to manage postpartum haemorrhage and decrease maternal morbidity. As part of the training, Lutgendorf et al. [20] included an assessment of PPH risk factors in patients in which the maternal team had to assess if the patient had placenta abruption, suspected or confirmed placenta praevia, multifetal gestation, or intrapartum use of magnesium sulphate. PPH risk factors were allocated for prompt identification and diagnosis during admission history taking and physical examination. Moreover, patients with two or three of the discussed risk factors in the antepartum or intrapartum period were classified as being at risk for PPH, and early intervention was implemented to avoid complications of PPH. In Tanzania, Egenberg et al. [13] conducted a quasi-experimental pre- and post-intervention study investigating the impact of multi-professional, scenario-based training on PPH. The training focused on technical and non-technical skills, such as interprofessional communication, when to call for help, decision-making in terms of assessment, diagnosis, and intervention for PPH, collaborative management, respective care, and information sharing with patients and relatives. Using realistic case scenarios of PPH, the intervention promoted critical thinking and clinical reasoning for prompt problem solving within a team during PPH. The training included reviewing PPH care bundles or guidelines, which allowed all interprofessional team members managing PPH to be prepared should PPH occur. The training further included debriefing sessions led by interprofessional facilitators, which assisted in solidifying roles and responsibilities, team cohesion, and team reflection on how to work best with each other and utilise each other’s expertise. In a post-review study based on the visual estimation of PPH, 0,9% of mothers were diagnosed with PPH before the training and 1.3% after training due to a gap in prior identification and documentation, which was then aided by the interprofessional intervention. A similar training program was conducted in Philadelphia, at the Pennsylvania Academic Medical Center, with 276 participants, including physicians, nurses, obstetricians, advanced practice nurses, and ancillary staff. The interprofessional intervention included didactic modules, interprofessional skills stations led by trained nurses, and in situ simulations. Post-assessment surveys indicated that participants rated the program 3.94 out of 4.00 for overall effectiveness in improving interprofessional team responses to PPH. The four key components of the obstetric haemorrhage safety bundle readiness, recognition, prevention, response, reporting system learning were used to aid in the prompt identification and diagnosis of PPH. In the United States, the California Maternal Quality Care Collaborative developed a comprehensive quality improvement toolkit for postpartum haemorrhage based on the national patient safety bundle for obstetric haemorrhage and noted positive outcomes in the early identification and diagnosis of PPH.

#### Fast administration of uterotonic medication and blood transfusion

A multimethod, quasi-experimental, pre-post intervention study by [28] revealed that hands-on training helped familiarise trainees with each step of the PPH procedure, which enabled them to retrieve skills automatically in a demanding emergency. Interprofessional collaborative practices, especially those implemented through simulation training, have been proven to be important in improving the administration of uterotonic medications and blood transfusions during PPH [29]. The same study’s results indicated a significant improvement in clinical responses to PPH after the implementation of a training program. Both [29] and [30] studies observed a notable reduction in the need for whole blood transfusions, suggesting better initial management of PPH, which potentially reduced the severity of bleeding episodes. Moreover [29], studies observed a decline in maternal deaths related to PPH from seven to four, indicating enhanced effectiveness in managing life-threatening haemorrhages through prompt and efficient coordinated care. In conclusion, providing uterotonic medication and blood transfusion swiftly in a case of PPH requires a coordinated interprofessional approach that includes an interprofessional team with various areas of expertise. Effective collaboration and continuous training of healthcare professionals ensure that every PPH patient receives the right care promptly during this critical period.

### Theme 2: Promotes patient safety and quality standards in managing PPH

The second theme that emerged from the study was that interprofessional collaborative practices promote patient safety and quality standards in PPH management. Below, the authors discuss the two subthemes.

#### Promotes adherence to clinical protocols for the management of PPH

The findings of a study on interprofessional simulation-based training in the prevention and management of PPH, conducted in a rural referral hospital in Northern Tanzania, revealed that compliance with clinical interventions for the management of PPH increased. The number of women who received 10 IU of oxytocin after training (91.7%) was significantly higher than that before training (87.8%). The proportion of women with PPH receiving 10 IU of oxytocin as part of the management of PPH significantly increased from 43.0% before training to 61.2% after training [31].

The authors further reported that after the interprofessional simulation-based training, a significantly greater percentage of women who received uterotonic drugs within one minute after birth was noted as 44.3% as compared to 40.4% noted before training, which is a requirement stipulated in the clinical protocol for managing PPH [29]. This was confirmed by Sageer et al. [30], whose findings revealed that before interprofessional obstetric simulation training on the management of PPH, 82% of deliveries received no medications for the treatment of haemorrhage, which decreased to 49% after training. The use of tranexamic acid (TXA) during deliveries also significantly increased from 2.72% to 4.76% after interprofessional simulation training [30]. The removal of the placenta by controlled cord traction and subsequent uterine massage was more frequently performed after training (98.8% and 99.0%, respectively) than before training (96.5% and 93.0%, respectively). The United States also revealed that the interprofessional collaborative practice intervention significantly improved knowledge for the obstetrics interprofessional team in managing PPH and adhering to PPH protocols by the obstetric, anaesthesia, and nursing team that manage PPH [32].

#### Positive outcomes

According to Wallace et al. [32], timely and effective team coordination optimises the response and improves clinical outcomes for PPH with team-based drills and haemorrhage bundles. Safety programs and bundles, including interprofessional simulation, protocols, and algorithms, have been associated with improved outcomes for PPH [32]. The findings of the studies conducted in Tanzania and Western Kenya revealed a significant improvement in the active management of the third stage of labour and management of PPH after the introduction of the interprofessional obstetric training programme, resulting in a marked decrease in PPH [33]. This is in line with previous reports that interprofessional simulation-based training has the best impact on decreasing severe PPH of more than 1500 ml and decreasing blood transfusions of more than 4 units. Literature also showed a reduction in maternal mortality after an interprofessional collaborative practice, thus interprofessional intervention to prevent maternity death promotes comprehensive patient care [12, 13, 33]. Based on the findings regarding interprofessional obstetric simulation training on the management of PPH, Zielinski et al. [34] shared the same sentiments, reporting a significant decrease in maternal morbidity, ranging from 6.35% before training to 5.28% thereafter. Additionally, the length of hospital stay of postpartum patients decreased from 2.05 to 2.01 days.

As a positive outcome, the review showed that interprofessional collaborative practices prevent complications associated with PPH. Brazil et al. [3] suggested that implementing interprofessional collaborative initiatives reduces probable complications from PPH and their sequelae, including emergency hysterectomy, resulting in a longer quality-adjusted life expectancy. Brazil et al. [3] affirmed similar findings in North and South Carolina and stated that the implementation of an interprofessional obstetric haemorrhage protocol led to an increase in the quantification of blood loss and a reduction in the number of women who experienced unplanned peripartum hysterectomies. Brazil et al. [3] also joined the discourse and elaborated that interprofessional collaborative practices provide safety tools to reduce unplanned hysterectomies and severe maternal morbidity through effective and comprehensive IPC.

### Theme 3: Enhances positive and effective interprofessional team collaboration in managing PPH

Interprofessional collaborative practices in the healthcare system are guided by principles of patient-centred care, a shared vision by the attending interprofessional team, role clarity, and accountable members who demonstrate effective interprofessional communication [7] The review has shown that effective IPC practices in the healthcare system result in clarity of roles and effective work processes among the team members that manage PPH emergency [7]. Awareness of the roles of various team members enables a shift workflow where there is no confusion or conflict when managing PPH [7]. A mixed method study in the United States, where nurses and obstetricians were involved in an interprofessional simulation study managing PPH, revealed that IPC had an immediate impact on improving team performance, and the team demonstrated an awareness of each other’s roles, displaying mutual respect and trust in each other’s clinical practice [24]. Effective IPC enables a patient safety culture and makes managing PPH a team-based effort, as the team works in alignment and according to set protocols [7]. A study in Denmark confirmed the above study and revealed that successful management of PPH is highly dependent on IPC efforts, where each healthcare professional is accountable for their role and responsibility when managing PPH [3].

#### Promotes relational coordination in the team that manages PPH

The second subtheme that emerged within this theme was that effective interprofessional collaborative practices promote relational coordination when managing PPH. In relational coordination when managing PPH, there needs to be a well-coordinated implementation of numerous tasks within a short period of time. Effective IPC from relational coordination enables good outcomes when managing PPH [10]. A multi-site before and after study was conducted in the United States across eight hospitals, where interprofessional simulation-based simulation training (Obs-STAT training) was implemented. The training was conducted on obstetrics team members who responded to obstetric emergencies, including but not limited to obstetrics, certified midwives, gynaecologists, and other health professionals. The IPC enabled the obstetrics team to be well coordinated when managing PPH, demonstrating improvement in coming quickly to the emergency site, acting on time and within their roles, and having closed-loop communication in the process [26]. In northern Tanzania in a qualitative study, the interprofessional team who attended the IPC programme in managing PPH stated that IPC improved their relational coordination and ability to use a teamwork approach effectively when managing PPH. Regardless of their job roles or tasks, the participants expressed that there was synergy in task allocation following the IPC intervention which enabled them to provide efficient PPH management for improved maternal and neonatal health [24]. Coordination among obstetrics teams managing PPH in Utah is critical because of the large number of births, approximately 3% of all births in the USA. A study in Utah by Brenneman et al. [10] affirmed the above, and the authors discussed that IPC has the potential to improve coordination which leads to the early referral and management of PPH.

#### Promotes accurate timelines of interprofessional communication among the obstetrics team

The last subtheme identified was the accurate timelines of interprofessional communication among obstetrics teams. According to Brenneman et al. [10], their collaborative action research study revealed that an IPC model for managing PPH promotes frequent communication among health professionals in different disciplines. Furthermore, the authors outline that the IPC model provides a deeper comprehension of teamwork and conflict resolution techniques to uphold effective interprofessional communication. Similarly, in a quality improvement project in California, similar results were obtained, promoting the IPC model for team cohesion. Singata-Madliki et al. [7] affirms that an IPC model in managing PPH reinforces positive interprofessional communication between the interprofessional obstetrics teams which creates a safe space for the team to self-correct and identify areas of improvement without prejudice or being hostile with each other.

## Discussion

This review emphasises the significance of early detection and diagnosis of PPH to reduce its severity. Studies have indicated that training programs using interprofessional simulation can provide healthcare professionals with the necessary skills to identify patients at risk of PPH early [6, 12–16]. Moreover, involving anaesthetic teams during the antepartum period has been recognised as a critical factor in proactively preparing and managing high-risk patients, potentially averting severe haemorrhagic complications. Hence, it is crucial to expand collaboration beyond obstetric personnel to include other members of the multidisciplinary team, such as physicians and pharmacists. A study conducted in Nigeria, Kenya, and South Africa found that training interprofessional obstetric professionals, including doctors and nurses, on blood loss estimation was essential for managing PPH and enhancing their skills. Furthermore, the review suggests utilising the clinical signs and symptoms of the patient along with weighing drapes and swabs for estimating blood loss [6]. Therefore, providing interprofessional training on blood loss estimation through collaboration and simulation in obstetric settings is pivotal for preventing and identifying PPH early. This study is affirmed by Singata-Madiki et al. [6] who encourage amending South African, maternity case records to include a place where the team can receive interprofessional training on PPH. Additionally, they recommend amending national maternity case records to include an observation document for recording cumulative blood loss at each time slot. In the absence of large-scale staffing which would permit the interprofessional team to assist in the recording of blood loss to avoid missing information for prompt diagnosis, health establishments would need to motivate sufficient staffing to perform the 15-minute post-delivery observation of vital signs and measurement of blood loss for prompt identification and diagnosis of PPH.

Subsequently, fast administration of uterotonic medication and blood transfusion is crucial for managing PPH. The above-mentioned interventions can help restore normal haemostasis and prevent complications. Moreover, the review revealed that there is a significant improvement in clinical outcomes, including a reduced need for whole blood transfusions and maternal deaths related to PPH, when uterotonic medication is promptly administered through the guidance and overall collaboration of a team in maternal care. Overall, the findings of this review highlight the critical role of IPC in promoting the prompt diagnosis and management of PPH.

Interprofessional collaborative practices have proven important for promoting compliance with clinical protocols for PPH management. Marked improvement in compliance following interprofessional simulation training has been noted. The findings revealed a marked increase in the routine administration of 10 IU of oxytocin to women post-delivery following training compared to before training. This symbolises the positive impact of interprofessional collaborative practices on the administration of post-delivery oxytocin. Improvement in the management of PPH was also marked, as evidenced by increased compliance in the administration of 10 IU of oxytocin as part of the management of interprofessional collaborative practices, such as interprofessional in situ simulation training. Not only did compliance increase in terms of drug administration, but the interprofessional collaborative simulation practices increased the time within which uterotonic drugs were administered, as healthcare professionals were able to administer uterotonic drugs within one minute. The implementation of protocols, such as the removal of the placenta by controlled cord traction and subsequent uterine massage, increased after interprofessional training, with positive results. The findings also revealed that the introduction of an interprofessional obstetric training programme led to cost-effectiveness, as there was a significant decrease in severe PPH, resulting in decreased blood transfusions. Patients’ length of stay in hospitals and maternal mortality were also reduced.

The positive outcomes of the interprofessional obstetric training program were not only seen in compliance with protocols and reduction in maternal mortality and blood transfusions, but positive interprofessional relationships and effective communication among professionals boosted their confidence regarding the management of PPH, consequently leading to a reduction in stress and anxiety and promotion of a positive psychological environment for the team. Acknowledgement and appreciation messages for improvements in PPH management and teamwork were ushered by postnatal women, their relatives, the community, and health authorities [***]. This reveals patient satisfaction and trust in professionals and facilities which is good for every healthcare establishment’s reputation. Moreover, the study revealed that effective IPC when managing PPH in the healthcare system enables an awareness of the roles and responsibilities of team members when managing PPH, and that this awareness is a prerequisite for successful management of the rapid and coordinated response by the nurses, obstetricians, and other healthcare professionals working under time-critical, high-risk, and emergency conditions such as PPH. Furthermore, the review showed that effective interprofessional collaborative practices promote relational coordination in a team when managing PPH. This competency enables the team to communicate effectively, collaborate well in teamwork, share leadership, and cope with stressful situations, and in the process, make use of safe clinical reasoning and problem-solving for positive patient outcomes.

### Recommendations

Interprofessional in situ simulation training programs/Standardized Obstetric simulation training and teamwork (OB-STAT) management of PPH was prevalent in the study as a recommendation for strengthening interprofessional collaborative practices in managing PPH. The E-MOTIVATE approach, whose objective is to measure blood loss every 15 minutes during the first hour after vaginal delivery to detect PPH early and trigger the initiation of first-line treatment and if necessary, escalate to advanced care. The training embeds the WHO guidelines and emphasises the need for teamwork, communication, collaboration, and shared values which are the core principles of interprofessional collaborative practices. Moreover, the above-mentioned training interventions promote interprofessional communication and awareness of roles in managing PPH through prompt diagnosis and management of PPH. Furthermore, this study revealed the importance of team-based consultation in managing PPH. Team-based consultation allows the sharing of expertise that can be conducted through interprofessional rounds and handover sessions with various healthcare professions.

In addition, safety bundles required for managing PPH should be developed by an interprofessional obstetrical team to include all important aspects of management. To socialise the interprofessional team to work effectively together in emergencies such as PPH, the study also recommends the use of standardised interprofessional semi-annual drills related to PPH with realistic scenarios, triggers, tasks to solve, and sufficient resources to practice in simulation. Simulation training facilitates the development of skills in a controlled environment where the patient is not injured. Subsequently, investments in relational approach training in managing PPH to promote mutual trust and respect, and culture-sensitive care among the team that manages PPH in efforts to minimise power dynamics and strengthen teamwork performance are recommended. Such training creates a positive psychological working environment in which the team functions well.

In the review, there were limited studies from an African/South African perspective that addressed the research question. Randomised control trial studies to measure the effectiveness of the interprofessional approach on patient outcomes when managing PPH as an interprofessional team can be conducted. Furthermore, for policy and national quality assurance in meeting the Sustainable Development Goals for decreasing maternal mortality and morbidity, the formulation of interprofessional maternal safety collaborative forums can be adopted by nations. The interprofessional forums can include but are not limited to obstetricians, pharmacists, physicians, and midwives to review state protocols and guidelines on maternal emergencies for public and private healthcare sectors for evidence-based practices. This recommendation is inspired by [31] the findings of interprofessional maternal safety collaborative forums.

## Conclusion

Interprofessional collaborative practices are essential for ensuring that maternal and child emergencies, such as postpartum haemorrhages, are managed effectively to ensure positive patient outcomes which reduce the occurrence of maternal morbidity. Interprofessional collaborative practices promote relational coordination, effective interprofessional communication, shared leadership, effective decision-making, and holistic, team-based interventions. This practice leads to the early diagnosis and management of postpartum haemorrhage. Not only is interprofessional collaborative practice good for the mother and child, but it also benefits the healthcare system by ensuring cost-effective care practices, where resources are utilised correctly, lessens hospital stay, and in this context, the patient experiencing postpartum haemorrhage receives a prompt blood transfusion and comprehensive management to prevent complications such as unplanned hysterectomies. Thus, quality and safe healthcare derived from interprofessional collaborative practice when managing postpartum haemorrhage is a necessity that must be intentionally strengthened in all healthcare settings. This practice can be socially introduced to all healthcare/medical professionals from undergraduate programmes when learning how to provide care during the third stage of labour through interprofessional simulation training or interprofessional team-based learning. Post-licensure progressive interprofessional initiatives should be explored, including childbirth assistance, to close any gaps due to lack of skill or knowledge when caring for a patient presenting with PPH. Therefore, this calls for a collaborative approach and a review of guidelines and policies to incorporate interprofessional collaborative practices into maternal care [35–48].

## Electronic supplementary material

Below is the link to the electronic supplementary material.

Supplementary Material 1

## Data Availability

No datasets were generated or analysed during the current study.
