# Supplementary material for: A scoping review on the contribution of interprofessional collaborative practices on preventing and managing post-partum haemorrhage in the health care system
Source: BMC Nurs. 2025 Apr 24;24:455. doi: 10.1186/s12912-025-02988-z (PMC12020204; doi:10.1186/s12912-025-02988-z)
Supplement: Supplementary file 1 — Supplementary Material 1 [file 12912_2025_2988_MOESM1_ESM.docx]

| Records removed *before screening*:  Duplicate records removed: (n =17)  Records identified from:  Databases (n = 461)  PubMed (n=381)  CINHAL (n= 07)  Medline (n= 51)  Scopus (n= 22)  **Identification**  Records screened:  (n =444)  Records excluded after reading title and abstract: (n = 414)  Reports assessed for eligibility:  (n = 30)  **Screening**  Reports excluded after reading full text (n=16)  For the following reasons:  Not addressing IPC (n = 14)  Wrong outcome (n=02)  Studies included in the review:  (n =14)  **Included**  **Figure 1: The PRISMA flow diagram describing the screening process is adapted from the PRISMA Group (2009) by Moher D, Liberati A, Tetzlaff J, Altman DG, and The PRISMA Group (2009). Preferred Reporting Items for Systematic Reviews and Meta-Analyses: The PRISMA Statement. PLoS Med 6(7): e1000097.**[**https://doi.org/10.1371/journal.pmed1000097**](https://doi.org/10.1371/journal.pmed1000097) |
| --- |
